# Protocol for a multicenter prospective cohort study evaluating arthroscopic and non-surgical treatment for microinstability of the hip joint

**DOI:** 10.1186/s12891-022-05269-x

**Published:** 2022-03-31

**Authors:** Axel Öhlin, Eric Hamrin Senorski, Mikael Sansone, Gretchen Leff, Neel Desai, Ida Lindman, Olufemi R. Ayeni, Marc R. Safran

**Affiliations:** 1 Department of Orthopaedics, University of Gothenburg, Gothenburg, Sweden; 2 Department of Health and Rehabilitation, University of Gothenburg, Gothenburg, Sweden; 3 Department of Physical Therapy, Stanford University, Redwood City, CA USA; 4 Division of Orthopaedic Surgery, Department of Surgery, McMaster University, Hamilton, ON Canada; 5 Department of Orthopaedic Surgery, Stanford University, Redwood City, CA USA

**Keywords:** Hip joint, Microinstability, Physical therapy, Arthroscopy

## Abstract

**Background:**

Microinstability of the hip joint is a proposed cause of hip pain and reduced function in young individuals. The underlying mechanism is thought to be extraphysiological hip motion due to bony deficiency and/or soft tissue deficiency or decreased soft tissue function. Recently, the condition has gained increased attention, and despite the fact that treatment today includes both non-surgical and surgical approaches, there is limited evidence on diagnostic specificity and treatment effects. The aim of this study is to evaluate clinical outcomes of both non-surgical and surgical treatment for microinstability of the hip joint.

**Methods:**

A multicenter prospective cohort study is planned to evaluating the outcome of physical therapy aimed at stabilizing the hip joint, as well as arthroscopic plication of the hip joint capsule, if the physical therapy fails. Outcomes will be evaluated using hip-specific patient-reported outcome measures: the short version of the International Hip Outcome Tool and the Copenhagen Hip and Groin Outcome Score, strength and function tests, health-related quality of life as determined using the European Quality of Life-5 Dimensions and the European Quality of Life-Visual Analog Scale, sports activity levels according to the Hip Sport Activity Scale, and reported complications. Patients will be evaluated at 6, 12 and 24 months after each treatment.

**Discussion:**

It is important to evaluate the clinical outcomes of both non-surgical and surgical treatment for suspected microinstability of the hip joint, and the planned prospective evaluation will contribute to the understanding of non-surgical as well as surgical treatment outcomes, including complications.

**Trial registration:**

Clinicaltrials.gov: NCT04934462. Registered June 22 2021.

**Supplementary Information:**

The online version contains supplementary material available at 10.1186/s12891-022-05269-x.

## Background

Microinstability of the hip joint is defined as extraphysiologic hip motion or pathological laxity that causes hip pain, with or without a subjective feeling of instability, and results in reduced hip function among young and active individuals [1–5]. The prevalence of hip microinstability is still unknown; however, the current data suggest that it is more common in women [6]. Hip stability depends on static and dynamic stabilizers. Static stabilizers comprise the bony morphology of the acetabulum and proximal femur, labrum, and capsular and non-capsular ligaments, with the iliofemoral ligament being the strongest ligament in the body. The negative intra-articular pressure, adhesion-cohesion, and muscle forces comprise the body´s dynamic stabilizers. Microinstability of the hip joint can be due to acetabular dysplasia, connective tissue disorders, macrotraumas, microtraumas, and iatrogenic and idiopathic causes [3]. Traditionally considered a highly stable joint, studies have shown that there is a range of translation in different hips, supporting the possible existence of hip microinstability [7–10]. In the setting of repetitive and/or forceful rotational or axial loading, this motion could stress the labrum, capsuloligamentous structures, and articular cartilage, ultimately leading to damage of the same. A unique pattern of articular cartilage damage, with inside-out abrasion, and labral- and chondral damage straight anteriorly and straight laterally has been shown to be associated with hip microinstability [11].

The diagnosis of hip microinstability is still poorly defined but is based on a combination of symptoms, physical examinations, and imaging findings. Patients rarely complain of hip instability; instead, deep pain in the hip joint, such as the c-sign where pain is felt deep in the inguinal crease or groin, is the predominantly reported symptom. Three physical examination tests used to identify microinstability have been evaluated: the abduction-hyperextension-external rotation (AB-HEER), the prone instability, and the hyperextension-external rotation (HEER) tests. The specificity for these tests ranges from 85.1–97.9%, and sensitivity ranges from 33.9–80.6% [12]. In addition to these physical examination tests, the range of motion (ROM) of the hip joint has been evaluated, and a combined flexion and rotation arc ≥200° has been associated with hip microinstability, with a sensitivity of 68.9% and specificity of 80.0% (Curtis., *in submission*). For imaging evaluation, the Cliff sign has been identified as a radiographic finding present in 89% of patients with hip microinstability, compared to 27% among patients without hip microinstability; in addition, the femoro-epiphyseal acetabular roof (FEAR) index has been used to distinguish between stable and unstable hips [13–15]. Moreover, magnetic resonance arthrography (MRA) findings associated with microinstability of the hip joint include anterior hip capsular thinning distal to the zona orbicularis (<3 mm) [16, 17]. Further suspicion of the diagnosis of hip microinstability can arise intraoperatively, demonstrating the ease of distraction of the hip joint together with visualization of the specific inside-out articular chondral wear pattern, perifoveal articular cartilage damage, and damage to the articular cartilage and/or labrum straight anteriorly or straight laterally (Safran et al., *in submission*)  [12, 18].

Treatment for hip microinstability is initiated with non-surgical treatment consisting of physical therapy that is mainly aimed at stabilizing the hip. If non-surgical treatment fails, surgery with arthroscopic plication of the hip joint capsule has yielded favorable results with low complication rates [6]. The anterior hip capsule has been shown to play an important role in hip stability, and in the applied arthroscopic technique, a plication of the capsule is performed in the region of the capsule that does not have ligamentous thickening. This is performed to tighten the hip capsule and improve the stability of the hip joint without limiting the hip’s motion or overconstraining the joint [19, 20].

An increasing body of evidence supports microinstability as a cause of hip pain; however, high-level scientific evidence supporting its role is still lacking. The current literature predominantly consists of retrospective and smaller prospective cohort studies [6, 21, 22]. It is of paramount importance that clinical decision-making is based on high-level of scientific evidence, as well as high methodological quality. The aim of the present prospective study is to evaluate the treatment outcomes of non-surgical and arthroscopic treatment for hip microinstability.

### Study objectives

The primary research objective is to evaluate the outcomes of non-surgical and arthroscopic treatments for hip microinstability using patient-reported outcome measures (PROMs) and strength and function tests up to two-years following treatment.

The secondary objectives are to evaluate health-related quality of life (HRQL), sports activity levels, and complications of non-surgical and arthroscopic treatment.

## Methods

### Study design

This is prospective cohort study will include patients ≥ 18 years of age. Patients will be evaluated clinically and radiographically at enrollment, and with PROMs and strength and functional tests at enrollment and at 6, 12, and 24 months following treatment. Patients will be recruited by experienced hip surgeons and sports medicine researchers at multiple national clinical sites. Hip function and HRQL will be evaluated using the short version of the International Hip Outcome Tool (iHOT-12), the Copenhagen Hip and Groin Outcome Score (HAGOS), the Hip Sports Activity Scale (HSAS), the European Quality of Life-5 Dimensions (EQ-5D), the European Quality of Life-Visual Analog Scale (EQ-VAS), and strength and functional tests [23–26]. Ethical approval for this study was granted by the Swedish Ethical Review Authority (#2020-05416). Written informed consent will be obtained from all the participants. This study complies with the declaration of Helsinki.

### Participant selection

#### Eligibility criteria

The inclusion criteria are as follows: 1) the patient experiences hip pain; 2) presence of at least one other finding suggestive of hip microinstability on physical examination or imaging; and 3) at least 50% reduction in pain on the Visual Analog Scale (VAS) following intra-articular hip injection of mepivacaine (10 ml, 10 mg/ml). The physical examination findings suggestive of hip microinstability that will be considered include a positive AB-HEER test, positive prone instability test, positive HEER test, Beighton score >4, and combined flexion and rotation arc ≥200°. The imaging findings suggestive of hip microinstability that will be considered are a borderline dysplasia (defined as a lateral center edge (LCE) angle of 20° to <25°, an anterior center edge (ACE) angle of 20° to <25°, and a Tönnis angle >10°–14°), a FEAR index > -5°, and a positive Cliff sign (Table 1). The LCE angle, Tönnis angle, FEAR index and Cliff sign will be evaluated using standardized anteroposterior (AP) view radiographs. The ACE angle will be evaluated using false-profile view radiographs. In addition, magnetic resonance imaging (MRI) will be performed to assess differential diagnoses. The exclusion criteria are as follows: 1) age <18 years; 2) Tönnis grade > 0 ; 3) LCE angle <20°; 4) ACE angle < 20°; 5) Tönnis angle >14°; 6) history of Legg–Calve–Perthes disease; 7) avascular necrosis of the femoral head; 8) prior hip surgery; 9) pigmented villonodular synovitis (PVNS); 10) synovial chondromatosis; and 11) other conditions that make the diagnosis of microinstability unlikely.

**Table 1:** Inclusion criteria

Symptoms	Hip pain
Physical examination	AB-HEER test
	Prone instability test
	HEER test
	Hip flexion + rotation arc ≥ 200°
	Beighton score >4
Imaging	FEAR index > -5°
	Borderline dysplasia • LCE 20° to <25° • ACE 20° to <25° • Tönnis angle >10° to 14°
	Cliff sign
Intra-articular hip injection (mepivacaine)	At least 50% reduction of pain

The inclusion criteria are symptoms of hip pain together with at least one other positive finding on physical examination or imaging suggestive of hip microinstability and at least a 50% reduction in pain following an intra-articular hip injection of Mepivacaine. *AB-HEER* abduction-hyperextension-external rotation, *ACE* anterior center edge, *HEER* hyperextension-external rotation, *FEAR* Femoro-Epiphyseal Acetabular Roof, *LCE* lateral center edge

#### Participant recruitment

Patients will be recruited from participating center clinics. Patients are mainly referred to these clinics by other orthopedic surgeons who do not specialized in hip preservation, as well as by family medicine doctors and physical therapists. All patients who fulfill the study criteria will be considered for inclusion and asked for their consent to participate.

### Study exposure

The study groups will be defined by the given treatment and divided into groups: the non-surgical treatment only group (group 1) and the failed non-surgical treatment followed by arthroscopic treatment group (group 2).

#### Non-surgical treatment

All patients (both groups 1 and 2) will undergo six months of non-surgical treatment in the form of physical therapy aimed at stabilizing the hip joint. The physical therapy will include exercises aiming to improve and strengthen the hip musculature (see Additional file 1).

#### Arthroscopic treatment

For patients with failed non-surgical treatment, which is defined as unsatisfactory hip function reported by the patient at 6-month follow-up, and where it is deemed that the patient could benefit from surgical treatment, diagnostic hip arthroscopy will be performed. The perioperative diagnostic criteria for hip microinstability are based on international expert consensus and are presented in Table 2 (Safran et al., *in submission*). If further signs of microinstability are found intraoperatively, arthroscopic treatment will be performed in the same session. Arthroscopic treatment of hip microinstability is performed with the patient in a supine position, on a traction table, with a well-padded perineal post. Standard hip arthroscopic portals are utilized, and plication of the capsule is performed with a suture shuttling technique in the region of the capsule that does not have ligamentous thickening, between the iliofemoral- and ischiofemoral ligaments [6]. Postoperatively, patients will be limited to foot flat weight bearing of no more than 10 kg in a hip orthosis, with their ROM limited to 0° to 90° of hip flexion for 2 weeks, and no supine straight leg raises for 4 weeks. Postoperative rehabilitation will follow the same rehabilitation protocol as that for non-surgically treated patients (see Additional file 1).

**Table 2:** Perioperative diagnostic criteria for hip microinstability

Ease of hip distraction under anesthesia
Inside out pattern of chondral damage
Location of chondral damage on the acetabulum (straight anterior or straight lateral)
Pattern of labral damage (labral chondral junction)
Anteroinferior labrum chondral damage
Perifoveal cartilage damage
Presence of a focal capsular defect
Capsular status (thin and poor quality)

### Study outcomes

The primary outcome is hip function determined by PROMs and strength and function tests undertaken 24 months after treatment. Secondary outcomes include HRQL, sports activity levels, and treatment complications.

PROMs for hip function include the iHOT-12 and HAGOS. The iHOT-12 consists of 12 questions covering symptoms and functional limitations, sports and recreational activities, and job-related as well as social, emotional, and lifestyle concerns. The HAGOS consists of 37 questions divided into six subscales: symptoms, pain, physical function in daily living, function in sports and recreational activities, participation in physical activities, and quality of life. Both the iHOT-12 and HAGOS have been translated and validated in Swedish and have shown sound psychometric properties when used for young and active individuals with hip complaints [27, 28].

Maximal isometric hip muscle force for hip flexion, extension, adduction, and abduction will be assessed using an externally fixated dynamometer or a handheld dynamometer (Hoggan MicroFET2, Hoggan, Scientific L.L.C., Salt Lake City, USA). The maximum developed force in Newtons (N) will be recorded.

Hop performance will be measured with three single-leg hop tests: vertical hop (Muscle lab, Ergotest Technology, Oslo, Norway), hop-for-distance, and 30-second side-hop test. Each hop test is performed with the patients holding their hands behind their backs. For the vertical hop, the time from takeoff to landing is converted into hop height in centimeters (cm). In the hop-for-distance test, the distance between the tip of the toes at takeoff to the heel at landing is measured in cm. For the 30-second side-hop test, one trial per leg is allowed, where the patient will be instructed to hop as many times as possible over two lines that are 40 cm apart. The number of hops will be recorded.

HRQL will be evaluated using the EQ-5D and EQ-VAS. Sports activity level will be determined via the HSAS, which is a hip joint-specific sports activity scale that ranges from no performance of recreational or competitive sport to performance of competitive sports at national and international elite levels. The HSAS has been translated and validated in Swedish and has shown sound psychometric properties when used with young and active individuals with hip complaints [29]. All complications, including, but not limited to, stiffness, continued instability, reoperations and or conversion to a total hip arthroplasty (THA), will be documented by researchers.

### Study follow-up

Patients will be followed-up for 24 months post-treatment (i.e. midterm follow-up). Group 1 will be followed-up 24 months after initiation of physical therapy, and group 2 will be followed-up 24 months after surgery. PROMs will be completed electronically by the patient at baseline, and at 6, 12, and 24 months post-treatment. Strength and function tests will be evaluated during in-person visits by physiotherapists at baseline, and at 6, 12, and 24 months post-treatment. Number of physical therapist visits will be summarized at 6 months post-treatment. Documentation of complications will be recorded at 24 months post-treatment. Non-responders will be reminded via email and telephone multiple times before being considered as lost to follow-up.

### Sample size calculation

Based on previous results of arthroscopic hip preservation surgery, for the comparison of pre- and post-treatment hip function, a minimum sample size of 26 patients for each treatment group is needed to reach a power of 80% with the iHOT-12 (sigma 25.5, alpha 0.05, effect size 20) [30].

### Statistical plan

Changes in hip function, sports activity levels, and HRQL at 6-, 12-, and 24-month follow-ups compared to baseline within groups will be evaluated using the Wilcoxon signed-rank test. Comparisons between groups 1 and 2 will be performed using the Mann–Whitney U test. The results will be reported as numbers, distribution values, and associated *p*-values. Complications will be reported as numbers and percentages. No interim analyses are planned. The alpha value will be set at <0.05.

### Study committee

The Steering Committee will provide guidance to the overall study and specific responsibilities of the committee include reviewing and approving the study protocol and resolve any challenges that arise during the study. The study committee have full authority how trial results should be communicated. Substantive contributions to the design, conduct, interpretation and reporting are required from authors on the final trial report.

### Data management

Demographic data, clinical and imaging findings, and procedures will be reported by the treating orthopedic surgeon. The PROM data will be reported by the patient using electronically administered questionnaires, and results from the strength and function tests will be reported by the treating physical therapist. Once received, the data will be visually checked by the researchers, and all missing, implausible, or inconsistent data will be queried. All data will be kept confidential and secure at the University of Gothenburg. All databases used for the storage of study data will be password-protected and accessible only to study personnel.

### Ethics

All patients who meet the eligibility criteria and are considered for inclusion will receive oral and written information and the option to ask questions regarding the study. Participation in the study is voluntary, and the patients can withdraw from the study at any time. Patients will have to sign a consent form before inclusion in the study. The study is performed according to the Helsinki Declaration and ethics approval for this study was granted by the Swedish Ethical Review Authority (#2020-05416).

## Discussion

The rationale for this study is as follows: 1) there is a need for more high-level scientific evidence on the treatment outcomes for hip microinstability; 2) the majority of existing studies have focused on hip-specific PROMs as outcomes, and objective data on strength and function are lacking; and 3) the current literature mainly evaluates surgical treatment, and more evidence on the outcomes of non-surgical treatment is needed.

This study is one of the few prospective cohort studies on the outcomes following treatment for hip microinstability. Strict diagnostic criteria and a thorough study design with prospective calculations of sample size, consecutive inclusion of patients, an appropriate follow-up time, means to reduce loss to follow-up, and adequate statistical analysis will contribute to its high methodological quality [31]. The use of not only suitable and validated hip-specific PROMs but also strength and functional tests, HRQL measures, and documentation of complications will provide a comprehensive evaluation of the treatment outcomes. A standardized rehabilitation protocol divided into different phases, with strict criteria for progression and clearly stated goals, will enable a worthwhile evaluation of non-surgical treatments and facilitate comparisons with surgical treatment.

With an increasing awareness of hip microinstability and a corresponding increase in both non-surgical and surgical treatment, high-level scientific evidence is needed to improve the given treatment and provide patients with realistic expectations. A prospective evaluation of treatment outcomes is needed to ensure that these often young and active patients are given a treatment that is effective and, simultaneously, does not expose them to unnecessary procedures and risks. This is of great importance not only for individual patients but also for the health care system.

## Supplementary Information

**Additional file 1.**

## Data Availability

Not applicable.

## Abbreviations

AB-HEER test: Abduction-hyperextension-external rotation test
ACE: Anterior center edge
AP: Anteroposterior
cm: Centimeter
EQ-5D: European quality of life-5 dimensions
FEAR index: Femoro-epiphyseal acetabular roof index
HAGOS: Copenhagen hip and groin outcome score
HEER: Hyperextension-external rotation
HRQL: Health-related quality of life
HSAS: Hip sports activity scale
iHOT-12: International hip outcome tool
LCE: Lateral center edge
MRA: Magnetic resonance arthrography
MRI: Magnetic resonance imaging
N: Newton
PVNS: Pigmented villonodular synovitis
PROM: Patient-reported outcome measure
ROM: Range of motion
THA: Total hip arthroplasty
VAS: Visual analog scale

## References

[1] Shu B, Safran MR (2011). Hip instability: anatomic and clinical considerations of traumatic and atraumatic instability. Clin Sports Med..

[2] Kalisvaart MM, Safran MR (2015). Microinstability of the hip-it does exist: etiology, diagnosis and treatment. J Hip Preserv Surg..

[3] Safran MR (2019). Microinstability of the hip-gaining acceptance. J Am Acad Orthop Surg..

[4] Jean P-O, Safran MR, Ayeni OR (2022). Hip microinstability: fact or fiction?. Knee Surg Sports Traumatol Arthrosc.

[5] Curtis DM, Murray IR, Money AJ, Pullen WM, Safran MR (2022). Hip microinstability: understanding a newly defined hip pathology in young athletes. Arthroscopy.

[6] Kalisvaart MM, Safran MR (2017). Hip instability treated with arthroscopic capsular plication. Knee Surg Sports Traumatol Arthrosc.

[7] d’Hemecourt PA, Sugimoto D, McKee-Proctor M, Zwicker RL, Jackson SS, Novais EN (2019). Can dynamic ultrasonography of the hip reliably assess anterior femoral head translation?. Clin Orthop Relat Res.

[8] Mitchell RJ, Gerrie BJ, McCulloch PC, Murphy AJ, Varner KE, Lintner DM (2016). Radiographic evidence of hip microinstability in elite ballet. Arthroscopy.

[9] Han S, Alexander JW, Thomas VS, Choi J, Harris JD, Doherty DB (2018). Does capsular laxity lead to microinstability of the native hip?. Am J Sports Med.

[10] Duthon VB, Charbonnier C, Kolo FC, Magnenat-Thalmann N, Becker CD, Bouvet C (2013). Correlation of clinical and magnetic resonance imaging findings in hips of elite female ballet dancers. Arthroscopy.

[11] Shibata KR, Matsuda S, Safran MR (2017). Is there a distinct pattern to the acetabular labrum and articular cartilage damage in the non-dysplastic hip with instability?. Knee Surg Sports Traumatol Arthrosc.

[12] Hoppe DJ, Truntzer JN, Shapiro LM, Abrams GD, Safran MR (2017). Diagnostic accuracy of 3 physical examination tests in the assessment of hip microinstability. Orthop J Sports Med.

[13] Packer JD, Cowan JB, Rebolledo BJ, Shibata KR, Riley GM, Finlay AK (2018). The cliff sign: a new radiographic sign of hip instability. Orthop J Sports Med.

[14] Wyatt M, Weidner J, Pfluger D, Beck M (2017). The Femoro-Epiphyseal Acetabular Roof (FEAR) Index: A new measurement associated with instability in borderline hip dysplasia?. Clin Orthop Relat Res.

[15] Truntzer JN, Hoppe DJ, Shapiro LM, Safran MR (2019). Can the FEAR Index be used to predict microinstability in patients undergoing hip arthroscopic surgery?. Am J Sports Med.

[16] Magerkurth O, Jacobson JA, Morag Y, Caoili E, Fessell D, Sekiya JK (2013). Capsular laxity of the hip: findings at magnetic resonance arthrography. Arthroscopy.

[17] Packer JD, Foster MJ, Riley GM, Stewart R, Shibata KR, Richardson ML (2020). Capsular thinning on magnetic resonance arthrography is associated with intra-operative hip joint laxity in women. J Hip Preserv Surg.

[18] Pullen WM, Curtis DM, Safran MR (2021). Central Femoral Head Chondromalacia Is Associated with a Diagnosis of Hip Instability. Arthrosc Sports Med and Rehabil.

[19] Johannsen AM, Behn AW, Shibata K, Ejnisman L, Thio T, Safran MR (2019). The role of anterior capsular laxity in hip microinstability - a novel biomechanical model. Am J Sports Med.

[20] Johannsen AM, Ejnisman L, Behn AW, Shibata K, Thio T, Safran MR (2019). Contributions of the capsule and labrum to hip mechanics in the context of hip microinstability. Orthop J Sports Med.

[21] Domb BG, Stake CE, Lindner D, El-Bitar Y, Jackson TJ (2013). Arthroscopic capsular plication and labral preservation in borderline hip dysplasia: two-year clinical outcomes of a surgical approach to a challenging problem. Am J Sports Med.

[22] Larson CM, Stone RM, Grossi EF, Giveans MR, Cornelsen GD (2015). Ehlers-Danlos syndrome: arthroscopic management for extreme soft-tissue hip instability. Arthroscopy.

[23] Griffin DR, Parsons N, Mohtadi NG, Safran MR, Multicenter Arthroscopy of the Hip Outcomes Research Network (2012). A short version of the International Hip Outcome Tool (iHOT-12) for use in routine clinical practice. Arthroscopy..

[24] Thorborg K, Holmich P, Christensen R, Petersen J, Roos EM (2011). The Copenhagen Hip and Groin Outcome Score (HAGOS): development and validation according to the COSMIN checklist. Br J Sports Med.

[25] Naal F, Miozzari H, Kelly B, Magennis E, Leunig M, Noetzli H (2013). The Hip Sports Activity Scale (HSAS) for patients with femoroacetabular impingement. Hip Int.

[26] Rabin R, de Charro F (2001). EQ-5D: a measure of health status from the EuroQol Group. Ann Med.

[27] Jonasson P, Baranto A, Karlsson J, Sward L, Sansone M, Thomee C (2014). A standardised outcome measure of pain, symptoms and physical function in patients with hip and groin disability due to femoro-acetabular impingement: cross-cultural adaptation and validation of the international Hip Outcome Tool (iHOT12) in Swedish. Knee Surg Sports Traumatol Arthrosc.

[28] Thomee R, Jonasson P, Thorborg K, Sansone M, Ahlden M, Thomee C (2014). Cross-cultural adaptation to Swedish and validation of the Copenhagen Hip and Groin Outcome Score (HAGOS) for pain, symptoms and physical function in patients with hip and groin disability due to femoro-acetabular impingement. Knee Surg Sports Traumatol Arthrosc.

[29] Öhlin A, Jónasson P, Ahldén M, Thomeé R, Baranto A, Karlsson J (2019). The Hip Sports Activity Scale for patients with femoroacetabular impingement syndrome - validation in Swedish. Transl Sports Med.

[30] Öhlin A, Sansone M, Ayeni OR, Swärd L, Ahldén M, Baranto A (2017). Predictors of outcome at 2-year follow-up after arthroscopic treatment of femoro-acetabular impingement. J Hip Preserv Surg..

[31] Slim K, Nini E, Forestier D, Kwiatkowski F, Panis Y, Chipponi J (2003). Methodological index for non-randomized studies (minors): development and validation of a new instrument. ANZ J Surg.

