# Supplementary material for: Protocol for a multicenter prospective cohort study evaluating arthroscopic and non-surgical treatment for microinstability of the hip joint
Source: BMC Musculoskelet Disord. 2022 Mar 31;23:309. doi: 10.1186/s12891-022-05269-x (PMC8973629; doi:10.1186/s12891-022-05269-x)
Supplement: Supplementary file 1 — Additional file 1. [file 12891_2022_5269_MOESM1_ESM.docx]

Additional file 1

# THE GOTHENBURG-STANFORD HIP MICROINSTABILITY REHABILITATION OUTLINE

This outline presents the suggested rehabilitation design for non-operative care for patients with hip microinstability. The outline is built on a criteria-based approach, with respect to time from symptom/injury/surgery.

## Non-OPerative Rehabilitation

| **Initial** | **Early stage** | **Mid stage** | **Late stage** |
| --- | --- | --- | --- |
| **General conditioning:**  - Cycling | **General conditioning:**  - Cycling  - Cross training  - Aqua jog (non-op)  - Walking | **General conditioning:**   - Cycling - Crosstraining - Rowing - Light running - Aqua Jog | **General conditioning:**   - Cycling - Crosstraining - Rowing - Running |
| **General strengthening**  Low load, high reps | **General strengthening**  Increasing load | **General strengthening**  High load, low reps | **General strengthening**  High load  Sport-specific |
| **Activation of Local Stabilizers**  **-**Rhythmic stabilization in quadruped or supine 90/90  -Seated hip ER  -Quadruped OKC hip ER (OKC)  -Quadruped CKC hip ER (knee push into table producing contralateral pelvic rotation)  -Quadruped Active hip flexion +/- resistance  - Hip flexor isometrics supine/sitting 90/90  **Lumbopelvic Stabilization**  -Movement control of pelvic motion in supine/sit/standing – AROM or clocking | **Progression of Local Stabilizers**  -Quadruped clamshell  -Quadruped Bird Dog +/- perturbations  -Sidelying hip ER (bottom leg- starting position in IR off edge of table with hip flexed) +/- load  -Heel slide to march with lumbopelvic stabilization  -Lower abdominal progression for gentle progressive hip flexor loading | **Progression of Local Stabilizers**  Standing progressions  -clamshell/Squat with flutter in deeper ranges of hip flexion  -Active Marching load – may progressively load if good form  -Standing mountain climber @ wall/sprinter start  Plank front/side  Pilates based ex appropriate here | **Progression of Local Stabilizers**  Farmer’s walk  Resisted skipping/marching  Pilates based ex appropriate here  Plank mountain climbers |
| **Movement Retraining**  *Focusing on “joint congruency” of hip- *most typically encouraging post glide of hip in setting of excessive anterior glide and minimizing hip hyperextension  Quadruped Rocking backward “hip hinge” with neutral spine (coxofemoral post glide)  -Apply hip hinge mechanics to bending/squatting/stairs/pt specific daily activities/athletic stance  *Introduce and focus on “symmetry” and symptom control  -Midrange control  -Minimize end range motions/rotations  -Respect structural barriers   - Standing Postures - Sitting posture - Gait - Stairs - Sport-specific functional training   (later stages) | **Dynamic control of hip to minimize anterior glide:**  Glut max retraining and minimize hamstring overuse  Glut isometrics (in slight hip flexion)  Heel squeezes (in slight hip flexion)  Hip ext with knee flexed  **Dynamic control of excessive superior/lateral glide (hip adduction)**  *Glut med retraining (OKC/CKC)  -Weight shifting in sagittal and frontal plane with focus on pelvofemoral control  -Hip abd isometrics at wall  -Walking sideways w/wo resistance  *Medial/Lateral balance (hip add/abd ratio)  -Pilates based exercises  -Standing ledge work hip abd/add | **Heavy strength training**  Squats  Deadlifts  Step-ups  Hip thrusters  Total hip  (maintain ROM control and modify after symptoms)  **Early Sport-Specific Training**  Accelerations  Decelerations  Multidirectional movements  Pilates | **Power and technique/Sport Specific as indicated**  Maximal sprints  Changes of direction  Cleans  Power-based strength training |

Abd, abduction; Add, adduction; AROM, active range of motion; CKC, closed kinetic chain; Ext, extension; Glut med, gluteus medius muscle; OKC, open kinetic chain; NM, neuromuscular; ROM, range of motion; WB, weightbearing

## Suggested progression Guidelines

Progress to next stage/progressive resisted global strengthening of hips as needed once patient demonstrates:

1) improved activation/control of local musculature without compensation,

2) improved arthrokinematics of hip, and

3) improved awareness of faulty movement patterns/postures

## Post-operative REHAB

Early post-operative precautions will be dictated by orthopaedic surgeon preference and surgical procedure, including but not limited to early ROM restrictions, WB restrictions, and/or use of brace. Considerations of precautions must be considered prior to implementation of interventions especially in the initial stages of rehab. To facilitate the assessment of progression, clinical goals have been determined for each phase of rehabilitation. Patients start their rehabilitation according the outlined post-operative stage below, before moving on to the stages of rehabilitation used for non-operative treatment.

Post-operatively patients will be limited to foot flat 10 kilogram weight bearing in a hip orthosis with ROM limited from 0 to 90 degrees of hip flexion for 2 weeks, and no supine straight leg raise for 4 weeks.

| **Post-operative stage** |
| --- |
| **Passive ROM:**  Within allowed ROM per Medical Doctor’s precautions  **WB/Gait training:**  per Medical Doctor’s precautions |
| **General strengthening**  Low load, high reps |
| **Muscle Activation/Active ROM**  *Stationary Bike (upright)  -Hip abd/ext/add/quad isometrics  -Prone hip IR AROM  -Prone lying  **Lumbopelvic Stabilization**  -Movement control of pelvic motion in supine/sit/standing – AROM or clocking  -Heel slides 🡺 30-70 degrees AAROM =>AROM with lumlbopelvic control |

## Goals Post-op

| **Initial/Post-op stage** | **Early stage** | **Mid stage** | **Late stage** |
| --- | --- | --- | --- |
| Decrease pain/normalize soft tissue mobility | Decrease pain | No pain | No pain |
| Walking without crutches with normalized gait pattern | Walking and cycling without pain | Running without pain | RTS |
| Full passive and active ROM | Strength recovery 75% | Strength recovery 80-85% | Full strength recovery (90%) |
| Appropriate muscle activation with anti-gravity movements OKC/CKC | Strength training without pain | Good LQ dynamic stability with unilateral CKC exercise/activities | Full hop and function recovery (90%) |

ROM, range of motion; RTS, return to sport

Strength testing will be performed for hip flexion, extension, abduction, and adduction.
